# Healthcare use in individuals with and without attention-deficit/hyperactivity disorder: A population-based longitudinal matched cohort study

**DOI:** 10.1371/journal.pmen.0000342

**Published:** 2025-07-28

**Authors:** Debra A. Butt, Ye Li, Rahim Moineddin, Braden O’Neill, Anthony D. Train, Jessica Gronsbell, Andrea S. Gershon, Karen Tu

**Affiliations:** 1 Department of Family and Community Medicine, Division of Research and Innovation, North York General Hospital, Toronto, Ontario, Canada; 2 Department of Family and Community Medicine, Scarborough General Hospital, Scarborough Health Network, Scarborough, Ontario, Canada; 3 Department of Family and Community Medicine, Temerty Faculty of Medicine, University of Toronto, Toronto, Ontario, Canada; 4 Department of Family Medicine, Queen’s Health Sciences, Queen’s University, Kingston, Ontario, Canada; 5 Department of Statistical Sciences, University of Toronto, Toronto, Ontario, Canada; 6 Sunnybrook Research Institute and IC/ES, Toronto, Ontario, Canada; 7 Department of Medicine, Temerty Faculty of Medicine, University of Toronto, Toronto, Ontario, Canada; 8 Toronto Western Family Health Team, University Health Network, Toronto, Ontario, Canada; University of Milano–Bicocca: Universita degli Studi di Milano-Bicocca, ITALY

## Abstract

Individuals with Attention-Deficit/Hyperactivity Disorder (ADHD) experienced worsening symptoms during the COVID-19 pandemic resulting in increased demand for healthcare services. However, it is unclear how those with and without ADHD utilized these services during the COVID-19 pandemic. This study examined healthcare utilization among individuals with and without ADHD and as a secondary objective, investigated these trends among female and male subgroups, from April 1, 2014-March 31, 2023. We conducted a population-based longitudinal retrospective cohort study among ADHD cases identified using a validated algorithm, and controls from Ontario, Canada over the same study period. We matched ADHD cases 1:1 to controls by sex, birth year, and geographical area. Outcomes were number of outpatient visits per person per fiscal year to family physicians, for mental health and to emergency departments, stratified by sex and age group over the follow-up period. Crude visit rate differences between sex-specific cases and controls were calculated with 95% confidence intervals (CI). We matched 427 716 ADHD cases to 427 716 controls. ADHD cases were 163 528 ≤ 17 years (32% female), and 264 188 adults (52% female). From 2013-2024, where March 17, 2020 marked the onset of the COVID-19 pandemic, females aged 1–17 years with ADHD appeared to have higher visit rate differences to family physicians, emergency departments, and increased mental health services, relative to their controls, particularly in 2020 [2.66 (95% CI: 2.65-2.68)]. In the same year, males with ADHD still had a higher mental health visit rate difference, [2.02 (95% CI: 2.01-2.02)] in 2020, but lower than that observed in females. Adult females with ADHD had the highest mental health visit rate difference in 2020 [5.09 (95% CI: 5.07-5.11)] and males with ADHD had 4.41 (95% CI: 4.40-4.43). These higher service utilization differences likely reflected greater health needs among females with ADHD while males underutilized these services.

## Introduction

Attention-Deficit/Hyperactivity Disorder (ADHD) is a common childhood-onset neurodevelopmental disorder worldwide, characterized by inattention, hyperactivity and/or impulsivity [1]. Prevalence estimates in children and youth have been increasing in Ontario, Canada from 2014-2021 with males having a cumulative rate of 9.59% and females having 5.26% in 2021 [2]. About 50–90% of childhood ADHD diagnoses may persist into adulthood [3–5]. ADHD is associated with other comorbid disorders involving the need for medical services: at least 40% of children and 80% of adults experience at least one comorbid psychiatric disorder [6–9] and are prone to accidental injuries and premature death [10]. The total socioeconomic cost of ADHD across the lifespan including both financial (healthcare, productivity, education, justice systems) and non-financial costs (disability) was estimated to be $12.76 billion US and $15,664 US per person in 2019 alone [11].

The demand for healthcare services pertaining to ADHD care has been steadily rising in recent years [2,12–18]. In Ontario, Canada, the heightened demand for ADHD-related visits in the primary care setting persisted during the COVID-19 pandemic in 2021 compared to the period prior to 2020, especially among female children and adults [12]. In the United States, the percentage of females with at least one stimulant prescription refill (primarily indicated for ADHD treatment) increased substantially by more than 10%, reflecting the average annual percent change from 2020-21, particularly in those aged 15–44 and 50–54 years [13]. The COVID-19 pandemic triggered widespread school closures worldwide [19–21] and was associated with lower detection rates and decreased referrals for new ADHD assessments [22,23]. In the United Kingdom, the pandemic exacerbated pre-existing strains on mental health services for ADHD due to longer wait times for care [23]. This reflected an escalation in ADHD symptoms in children and adults worldwide associated with the pandemic [12,24–31] but little is known about the pattern of healthcare use during this time and at the population level in different outpatient settings among those with and without ADHD in Canada.

A systematic review and meta-analysis involving 32 studies worldwide found that ADHD in children was associated with higher healthcare service use and costs compared to children without ADHD [32]. A cross-sectional retrospective chart abstraction of family physicians’ medical records conducted in Ontario, Canada for children and youth aged 1–24 years with ADHD (536 cases) found that they had a higher frequency of visits to family physicians for mental health concerns, and more psychiatrist and pediatrician visits compared to same-aged patients without ADHD [33]. These studies are mainly limited to children [32,34–36] and there are few population-level studies examining healthcare utilization in those with ADHD. In addition, healthcare utilization studies are mainly 1–2 decades old [9,36–41] and have not used validated case definitions to define ADHD [9,35,36,40].

Furthermore, there is a lack of information on the patterns of healthcare utilization among females and males with ADHD over time [9,32,36,37,39–41]. Prior studies have demonstrated sex differences in help seeking behaviour in the general population, particularly among female adolescents seeking more help for mental health concerns compared to males [42,43]. With the COVID-19 pandemic, there were significantly higher rates for mental health outpatient visits among female children, adolescents and young adults in the general population compared to expected patterns prior to the pandemic, while males have had lower mental health service use [44–46]. In light of these findings, it is important to ensure that females and males with ADHD have equal access to detection, diagnosis and management as misdiagnosis or late diagnoses of ADHD can lead to the adverse consequences associated with untreated ADHD and its comorbidities, including a reduced life expectancy [10,47]. Therefore, the aim of this study is to examine trends in healthcare utilization to family physicians, for mental health from any physician, and to emergency departments among individuals with and without ADHD from 2014-2023. The secondary objective of this study is to examine these trends in females and males with and without ADHD. By investigating healthcare utilization as an indicator of ADHD symptoms in both females and males, we can begin to formulate strategies to improve the well-being of individuals with ADHD and thereby lower the socioeconomic burden of the disorder.

## Methods

### Study design and setting

We conducted a population-based, longitudinal retrospective matched cohort study in Ontario, Canada from April 1, 2014 to March 31, 2023, where March 17, 2020 marked the onset of the COVID-19 pandemic [19]. Ontario is Canada’s most populous province with 15.5 million people as of April 1, 2023 (40% of the Canadian population) [48]. Ontario has a publicly funded health care system where all permanent residents are fully insured and eligible for universal health coverage under the Ontario Health Insurance Plan (OHIP). The Ontario Health Data Platform provided de-identified, anonymous, and privacy-protected data for this study via linkage to several large health administrative databases on a secure platform. In this study, the Strengthening the Reporting of Observational Studies in Epidemiology Statement [49] and the Reporting of studies Conducted using Observational Routinely-collected health Data statement were followed [50]. The North York General Hospital Research Ethics Board approved the study (0256–423).

### Data sources

We used health care administrative databases in Ontario linked through unique encoded identifiers which included OHIP to identify billing claims for physician services, Registered Persons Database for patient demographics such as sex and age, Canadian Institute for Health Information Discharge Abstract Database for clinical information on hospital admissions, National Ambulatory Care Reporting System to identify patients who presented to emergency departments and the Narcotic Monitoring Database for drug information on ADHD-specific medications such as stimulants.

### Cohort selection

The study population included all eligible Ontario residents aged ≥1 year registered with a valid health card from Aprill 1, 2014 to March 31, 2023. In the same time period, cases were identified using a validated health administrative case finding algorithm for ADHD defined as “2 physician visits for ADHD in 1 year or 1 ADHD-specific prescription” (sensitivity 83%, specificity 99%, positive predictive value 79% and negative predictive value 99%) [2]. The date of identification for the ADHD case, also called the index date, is the same assigned date for the identification of the matched control. Each case (ADHD patient) was matched to a control (without ADHD) by sex, birth year and living within the same residential area (dissemination area). Dissemination area is the smallest geographic unit representing an average population of 400–700 persons in Ontario reported by census data having the same socioeconomic status and rurality indices [51].

### Outcome

The primary outcomes were defined as the number of outpatient visits per person per fiscal year: i) for family physicians, ii) for mental healthcare and iii) for emergency department use. Fiscal year was defined from April 1^st^ of the current year to March 31^st^ of the following year. The outcomes for the ADHD cases and matched controls were calculated as the number of outpatient visits and measured yearly from the index date to the end of the fiscal year, loss of follow-up or death, whichever occurred first. We calculated the crude rates of the outpatient visits per fiscal year by dividing the number of outpatient visits by the person-year. We used primary care OHIP service codes and billing claims to determine family physician visits (S1 Appendix). To determine a mental health-related outpatient visit to any physician, we used mental health claims from OHIP that refer to physician billings associated with diagnostic codes that have been validated for mental health use in Ontario health administrative data [52] (S2 Appendix). To capture emergency department use, we used the National Ambulatory Care Reporting System database to identify all-cause visits using the International Classification of Diseases Code 10^th^ revision codes which excluded transfers, scheduled and elective visits.

### Covariates

We obtained age and sex from the Registered Persons Database; socioeconomic status was defined by neighbourhood income quintiles and rurality indices, representing rural and urban residence, were derived from the dissemination area. Neighbourhood income quintiles reflect average family income, adjusted for both household and community size. Patient comorbidity burden was determined using the John Hopkins Adjusted Clinical Groups System Version 13 to capture aggregated diagnosis groups and resource utilization band which are validated measures of comorbidity [53]. Aggregated diagnosis groups and resource utilization band were obtained by using hospital admissions from Canadian Institute for Health Information Discharge Abstract Database and OHIP claims within the 2-year period preceding the index date (date of first ADHD diagnosis).

### Statistical analysis

Descriptive statistics were used to summarize baseline patient demographic characteristics for cases and controls. Standardized differences were calculated to assess case and control differences. Absolute standardized differences ≥0.1 are generally considered a meaningful difference between groups [54]. Outcomes were stratified by sex (female/male) and age group (1–17 years to reflect children and adolescents/ ≥ 18 years to reflect adults) from 2014-2023. The 95% confidence intervals for rates were estimated assuming Poisson distribution. For ADHD cases and their controls, we plotted the rates of outpatient visits per fiscal year stratified by sex and age groups to visually assess the trends and visit rate differences for healthcare utilizations. Visit rate differences are defined as the differences in healthcare utilization between individuals with ADHD and their sex-matched controls. Subjects who moved to another age group during the study period were accounted for in our longitudinal study by using age group as a time-varying co-variate. We performed sensitivity analyses for each outcome examining visit rate differences for females and males with ADHD across the following age groups: 1–9 years, 10–17 years, 18–29 years, 30–49 years and aged ≥50 years. Data processing and analyses were performed using R 4.3.1 software on Ubuntu 18.04.

## Results

**Fig 1:**
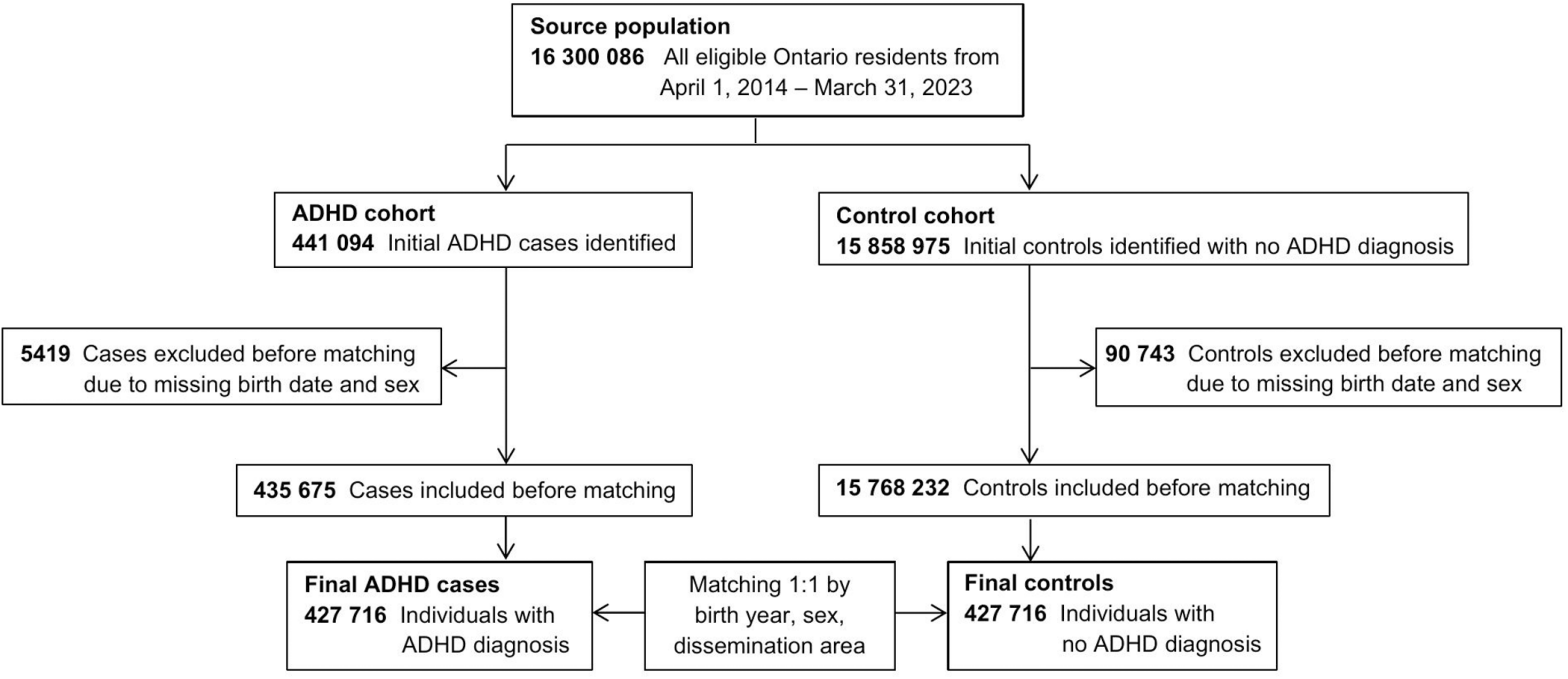
Flowchart of the cohort selection process. ADHD = Attention-Deficit/Hyperactivity Disorder; dissemination area = smallest geographic unit representing an average population of 400-700 persons in Ontario reported by census data.

This longitudinal retrospective cohort study involved 427 716 individuals with ADHD (cases) matched 1:1 with individuals without ADHD (controls) from April 1, 2014 to March 31, 2023 (Fig 1).

There were 163 528 children and adolescents aged 1-17 years with ADHD with a mean (standard deviation) age of 10.4 (3.8) years where 32.5% were females. There were 264 188 adults aged 18 years and older with ADHD with an average age of 35.1 (14.4) years where 52.4% represented females. Table 1 details demographic characteristics among the ADHD cases and controls indicating higher urban residence (86%) and balanced income distributions among cases and controls. There were unbalanced groups among the overall ADHD cases and controls as indicated by the standardized differences for the aggregated diagnosis groups and resource utilization band (Table 1). Among those with aggregated diagnosis groups’ scores ≥5 diagnoses, females with ADHD had the highest proportion of comorbidities. Among those with resource utilization band scores 4–5, females with ADHD also had the highest proportion of resource use (Table 1).

**Table 1:** Characteristics of ADHD cases and matched controls.

	Cases				Controls			Absolute Standardized Difference (Overall)
	Females	Males	Overall	Females		Males	Overall	
**Age**								
Mean (SD)	28 (16)	23 (17)	25 (17)	28 (16)		23 (17)	25 (17)	0.00
Median [Min, Max]	25 [1, 99]	19 [1, 98]	22 [1, 99]	25 [1, 99]		19 [1, 98]	22 [1, 99]	0.00
**Age Groups (years)**	No.(%)	No.(%)	No.(%)	No.(%)		No.(%)	No.(%)	
**1-9**	23,156 (11.5)	62,067 (27.4)	85,223 (19.9)	23,156 (11.5)		62,067 (27.4)	85,223 (19.9)	0.00
**10-17**	34,657 (17.2)	43,648 (19.3)	78,305 (18.3)	34,657 (17.2)		43,648 (19.3)	78,305 (18.3)	0.00
**18-29**	64,494 (32.1)	53,920 (23.8)	118,414 (27.7)	64,494 (32.1)		53,920 (23.8)	118,414 (27.7)	0.00
**30-49**	57,027 (28.4)	48,293 (21.3)	105,320 (24.6)	57,027 (28.4)		48,293 (21.3)	105,320 (24.6)	0.00
**50 and older**	21,780 (10.8)	18,674 (8.2)	40,454 (9.5)	21,780 (10.8)		18,674 (8.2)	40,454 (9.5)	0.00
**Rurality**	No.(%)	No.(%)	No.(%)	No.(%)		No.(%)	No.(%)	
**Rural**	27,484 (13.7)	31,951 (14.1)	59,435 (13.9)	27,484 (13.7)		31,951 (14.1)	59,435 (13.9)	0.00
**Urban**	173,630 (86.3)	194,651 (85.9)	368,281 (86.1)	173,630 (86.3)		194,651 (85.9)	368,281 (86.1)	0.00
**Neighbourhood Income Quintile**	No.(%)	No.(%)	No.(%)	No.(%)		No.(%)	No.(%)	
**1 (Lower)**	44,735 (22.2)	48,489 (21.4)	93,224 (21.8)	44,735 (22.2)		48,489 (21.4)	93,224 (21.8)	0.00
**2 (Lower-Middle)**	39,725 (19.8)	43,454 (19.2)	83,179 (19.4)	39,725 (19.8)		43,454 (19.2)	83,179 (19.4)	0.00
**3 (Middle)**	37,120 (18.5)	42,234 (18.6)	79,354 (18.6)	37,120 (18.4)		42,234 (18.6)	79,354 (18.6)	0.00
**4 (Middle-Higher)**	36,564 (18.2)	42,362 (18.7)	78,926 (18.5)	36,564 (18.2)		42,362 (18.7)	78,926 (18.5)	0.00
**5 (Higher)**	42,958 (21.4)	50,043 (22.1)	93,001 (21.7)	42,958 (21.4)		50,043 (22.1)	93,001 (21.7)	0.00
**Missing**	12 (<0.1)	20 (<0.1)	32 (<0.1)	12 (<0.1)		20 (<0.1)	32 (<0.1)	0.00
**Aggregated Diagnosis Groups**	No.(%)	No.(%)	No.(%)	No.(%)		No.(%)	No.(%)	
**0-4 (Low level of comorbidity)**	74,074 (36.8)	116,138 (51.3)	190,212 (44.5)	119,601 (59.5)		166,686 (73.6)	286,287 (66.9)	0.46
**5-9 (Middle level of comorbidity)**	96,908 (48.2)	93,083 (41.1)	189,991 (44.4)	68,929 (34.3)		54,116 (23.9)	123,045 (28.8)	0.33
**10+ (High level of comorbidity)**	30,132 (15.0)	17,381 (7.7)	47,513 (11.1)	12,584 (6.3)		5,800 (2.6%)	18,384 (4.3)	0.26
**Resource Utilization Band**	No.(%)	No.(%)	No.(%)	No.(%)		No.(%)	No.(%)	
**0 (Nonuser)**	10,290 (5.1)	15,333 (6.8)	25,623 (6.0)	30,432 (15.1)		45,405 (20.0)	75,837 (17.7)	0.37
**1 (Healthy user)**	1,993 (1.0)	4,603 (2.0)	6,596 (1.5)	9,741 (4.8%)		18,304 (8.1)	28,045 (6.6)	0.26
**2** (**Low user)**	22,998 (11.4)	44,316 (19.6)	67,314 (15.7)	40,117 (19.9)		62,831 (27.7)	102,948 (24.1)	0.21
**3 (Moderate user)**	109,059 (54.2)	121,219 (53.5)	230,278 (53.8)	85,111 (42.3)		82,329 (36.3)	167,440 (39.1)	0.30
**4 (High user)**	44,260 (22.0)	30,645 (13.5)	74,905 (17.5)	31,009 (15.4)		14,260 (6.3)	45,269 (10.6)	0.20
**5 (Very high user)**	12,514 (6.2)	10,486 (4.6)	23,000 (5.4)	4,704 (2.3)		3,473 (1.5)	8,177 (1.9)	0.19

Absolute standardized difference refers to overall cases and controls; Fiscal year = starts on April 1st and ends on March 31st of the following year; Max = maximum value; Min = minimum value; SD = standard deviation.

### Outpatient visits

**Fig 2:**
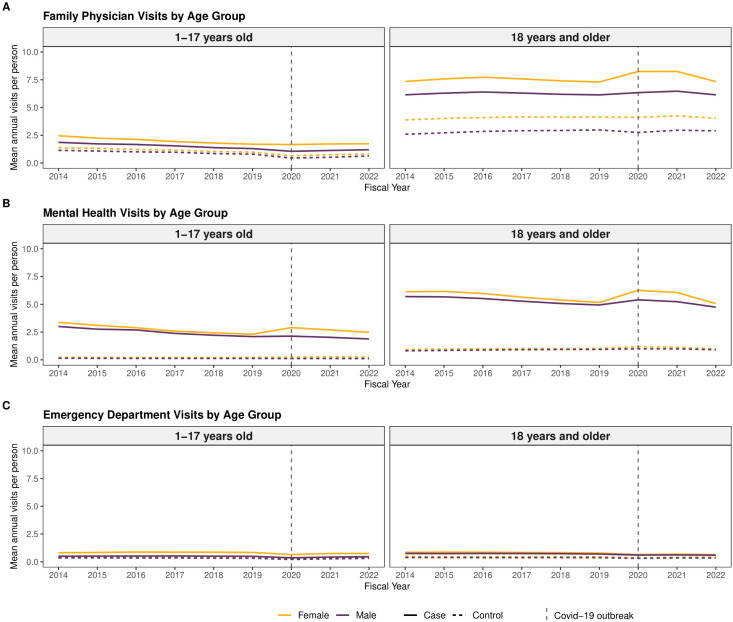
Mean annual visit rates among female and male cases and controls. Data for Fig 2 are found in the Supporting information file, see S1 Tables.

Fig 2 depicts the mean annual outpatient visits for fiscal years 2014–2022 among females and males with ADHD and their corresponding controls.

In 2020, both females and males aged 1–17 years with ADHD appeared to have fewer family physician visits/person (females: 1.66; 95% CI: 1.65-1.67, males: 1.06; 95% CI: 1.05-1.06) compared to the pre-pandemic period (Fig 2A and S1 Tables). Conversely, female and male adults with ADHD appeared to have increased family physician visits in 2020 at 8.24 (95% CI: 8.22-8.26) visits/person and 6.34 (95% CI: 6.33-6.36) visits/person, respectively (Fig 2A and S1 Tables).

Regarding mental health visits, females and males aged 1–17 years with ADHD appeared to have higher visits in 2020 relative to controls [females (2.90; 95% CI: 2.89-2.91; males (2.13; 95% CI: 2.13-2.14)]. Mental health visits peaked in 2020 for adult females with ADHD at 6.26 (95% CI: 6.25-6.28) visits/person and at 5.40 (95% CI: 5.39-5.42) visits/person for adult males (Fig 2B and S1 Tables).

Although annual emergency department visits were much lower than the other outpatient visits, females with ADHD tended to have higher annual emergency department visits relative to controls (Fig 2C and S1 Tables).

### Visit rate differences

**Fig 3:**
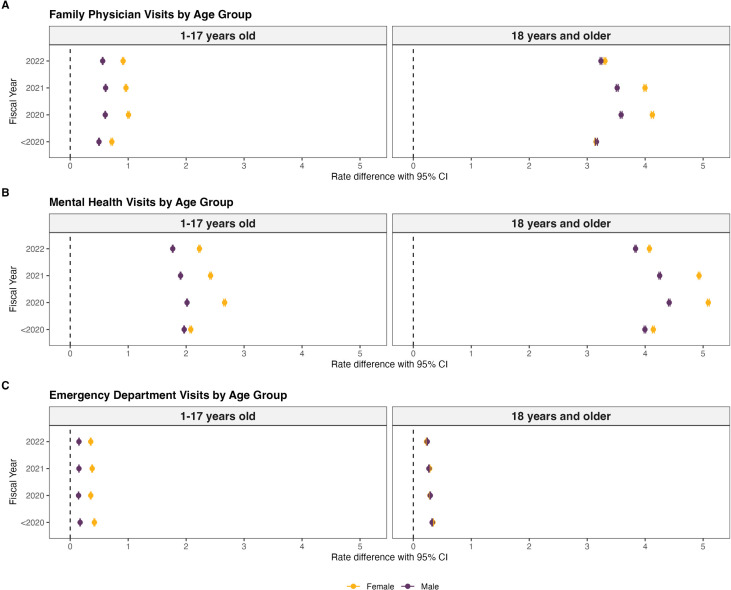
Visit rate differences among females and males with ADHD by age groups. Data for Fig 3 are found in the Supporting information file, see S1 Tables.

Fig 3 presents visit rate differences among sex-specific cases relative to matched controls, prior to the onset of the COVID-19 pandemic designated as <2020 from April 1, 2014-March 31, 2020, and annually during the COVID-19 pandemic from April 1, 2020-March 31, 2023.

#### Prior to the onset of the COVID-19 pandemic.

Females aged 1–17 years with ADHD tended to have higher family physician visit rate differences at 0.72 (95% CI: 0.70-0.73) and lower visit rate differences were observed in males aged 1–17 years with ADHD at 0.50 (95% CI: 0.49-0.51) (Fig 3A and S1 Tables). However, adult females and males with ADHD had similar family physician visit rate differences (Fig 3A and S1 Tables). Females with ADHD also appeared to exhibit slightly greater rate differences in mental health visits and we observed lower visit rate differences among males [female children and adolescents: 2.08 (95% CI: 2.07-2.09); male children and adolescents, 1.97 (95% CI: 1.96-1.97); female adults: 4.14 (95% CI: 4.13-4.16); male adults, 4.00 (95% CI: 3.98-4.01) (Fig 3B and S1 Tables). Females aged 1–17 years with ADHD appeared to have a higher emergency department visit rate difference at 0.42 (95% CI: 0.41-0.43) and lower visit rate differences were observed in males aged 1–17 years with ADHD at 0.17 (95% CI: 0.17-0.18); however, visit rate differences to the emergency department were similar in both adult females and males with ADHD (Fig 3C and S1 Tables).

#### During the COVID-19 pandemic.

Females aged 1–17 years with ADHD appeared to have higher family physician visit rate differences for fiscal years 2020–2022, where females had 1.01 (95% CI: 0.99-1.02) visits/person and males aged 1–17 years with ADHD had 0.60 (95% CI: 0.60-0.61) visits/person in 2020 (Fig 3A and S1 Tables). This trend in visit rate differences from 2020-2022 appeared somewhat similar among adults with ADHD, where females had 4.12 (95% CI: 4.10-4.15) visits/person and males had 3.59 (95% CI: 3.57-3.60) visits/person in 2020 (Fig 3A and S1 Table).

Similarly, females with ADHD appeared to have higher mental health visit rate differences during the pandemic (Fig 3B). In 2020, females aged 1–17 years with ADHD had a mental health visit rate difference of 2.66 (95% CI: 2.65-2.68) visits/person and males had 2.02 (95% CI: 2.01-2.02) visits/person, while adult females had 5.09 (95% CI: 5.07-5.11) visits/person and males had 4.41 (95% CI: 4.40-4.43) visits/person (Fig 3B and S1 Tables). Of note, from 2014-2022, females aged 1–17 years with ADHD appeared to consistently have higher emergency department visit rate differences (Fig 3C and S1 Tables). However, emergency department visit rate differences appeared similar among adult females and males with ADHD from 2020-2022.

### Sensitivity analysis

We conducted sensitivity analyses for outpatient visit rate differences in females and males with ADHD for the following age groups: 1–9 years, 10–17 years, 18–29 years, 30–49 years and aged ≥50 years. We observed that sex-specific visit rate differences changed before and during the COVID-pandemic for only those aged 30–49 years (S1 Fig and S2 Tables). Before the COVID-19 pandemic, males aged 30–49 years with ADHD consistently had higher outpatient visit rate differences and we observed lower visit rate differences for females: family physician visits [males, 3.88 (95% CI: 3.85-3.91); females, 3.45 (95% CI: 3.42-3.48)], mental health visits [males, 4.88 (95% CI: 4.85-4.91); females, 4.58 (95% CI: 4.56-4.61)], and emergency department visits [males, 0.38 (95% CI: 0.37-0.39); females, 0.34 (95% CI: 0.33-0.35)] (S1 Fig and S2 Tables). During 2020–2021, females aged 30–49 years appeared to have higher mental health visit rate differences, but by 2022, males again had the higher outpatient visit rate differences (S1 Fig and S2 Tables). We also observed that throughout the entire study period from 2014-2023, females aged 10–17 years with ADHD appeared to consistently have the highest emergency department visit rate differences (S1 Fig and S2 Tables).

## Discussion

To our knowledge, this is the first study to present descriptive trends on outpatient healthcare visits for both females and males with and without ADHD, stratified by specific age groups over a 9-year period. This longitudinal retrospective matched cohort study (2014–2023) found that female children and adolescents with ADHD appeared to consistently have higher average visit rate differences to family physicians and to emergency departments, with a substantial increase for mental healthcare in 2020. We observed a somewhat similar pattern for adult females with ADHD, except for those aged 30–49 years. While adult females with ADHD also appeared to have higher family physician visit rate differences, this trend was observed from 2020 onward.

Furthermore, we observed that female children and adolescents with ADHD, especially those aged 10–17 years appeared to have the highest emergency department visit rate differences, more so than any other age group throughout the entire study period. There are no other published studies demonstrating that female children and adolescents with ADHD have higher annual emergency department visit rate differences with respect to males over a 9-year period. A systematic review about children and adolescents with ADHD found only two out of the 32 included studies provided sex-stratified analyses on health service use in the United States [32,55,56], where male children and adolescents with ADHD, identified using diagnostic codes, had more emergency department visits compared to females during 2011–2012 [55]. Also, the United States National Ambulatory Medical Care Survey showed that male children and adolescents with ADHD had more than double the average annual physician office visits compared to females from 2012-2013 [57].

In the first year of the COVID-19 pandemic, we observed substantial increases in family physician visits for adult females and males with ADHD and a greater rise in mental health visits for both females and males with ADHD irrespective of age, compared with controls. Visits to family physicians in children and adolescents with ADHD, and to emergency departments for all individuals with ADHD decreased in 2020. Other studies have found conflicting results. A study conducted in the United States involving 8121 adolescents receiving mental health care from January 1, 2019 to December 31, 2021 found immediately after the onset of the pandemic, the rate of mental health visits for ADHD decreased by 14.5 mean visits per week compared to the pre-pandemic period but this trend was not persistent during the pandemic [58]. Another study done in the United States involving a community-based pediatric health care network from January 2016-March 2021 found ADHD-related visits in children decreased 33% in the first quarter (Q1) and then returned to expected rates by Q2-Q4; however, there was an increased proportion of female patients with first ADHD diagnosis during the first year of the pandemic [59]. Unlike this study, the aforementioned studies were not population-based, and ADHD-related visits were identified by diagnostic codes only which may not accurately capture individuals diagnosed with ADHD [58,59].

The diagnostic rates for females and males with ADHD are also changing over time. Historically, prevalence rates for ADHD with respect to sex differences have indicated 3–16 males for every one diagnosed female [60], decreasing in adulthood [60,61]. In Ontario, Canada, females with ADHD aged 1–24 years were estimated to have a 1.8-fold increase in prevalence rates from 2014-2021 [2]. Also, there were higher incidence rates for females with ADHD aged 1–24 years compared to males in 2021, where females demonstrated a 2.7-fold greater increase in incidence rates from 2014-2021 [2]. These findings lend further support to this study where females with ADHD appeared to use greater outpatient health services over time, and especially during the COVID-19 pandemic from 2020 to 2022. Another Ontario study demonstrated that the use of physician-based outpatient mental health services in Ontario, Canada rose for female children and adolescents following the first 18 months of the COVID-19 pandemic where females new to care used 22%, and those with pre-existing psychiatric conditions used 10% more mental health services compared to the pre-pandemic period [62]. In another population-based study in Korea, female children and adolescents with ADHD identified using diagnostic codes had a higher mean number of psychiatric visits during the COVID-19 pandemic versus the pre-pandemic period [63]. In a Swedish population-based matched case-control study that used diagnostic codes and/or ADHD medications to identify patients from January 1, 2010 to December 31, 2021, females with ADHD were more likely than males with ADHD to have both in- and outpatient mental and non-psychiatric visits 2 and 5 years after the ADHD index date defined as the diagnosis date or filling of an ADHD prescription [47]. These international studies suggest real differences in outpatient healthcare visits between females and males with ADHD.

Moreover, in our sensitivity analysis where we examined other specific age groups, we observed that sex-specific visit rate differences changed before and during the COVID-19 pandemic for only those aged 30–49 years with ADHD, which represented a total of 105 320 ADHD patients. Before the COVID-19 pandemic, males aged 30–49 years with ADHD appeared to consistently have higher visit rate differences to family physicians, for mental health and to emergency departments. During the COVID-19 pandemic, females with ADHD appeared to demonstrate slightly higher family physician visit rate differences and even greater differences for mental health visits, but by 2022, the pattern shifted back to males. This finding, prior to the COVID-19 pandemic, may reflect the increased health seeking behaviour of males with ADHD in adulthood who present to the healthcare system later in life due to an inability to cope with their illness. Danielson et al. (2023) reported a significant rise in ADHD prescription medications among older adult females with ADHD during the COVID pandemic from 2020 to 2021 [13] which is consistent with our study findings of increased mental health visits in females with ADHD. In a longitudinal matched cohort study conducted in Sweden, males with a first time diagnosis of ADHD between the ages of 30–45 years had similar healthcare utilization compared to same-aged females with ADHD for psychiatric outpatient visits; however, females tended to have greater psychiatric medication prescriptions than males [64]. Despite these findings, research examining sex differences in healthcare utilization patterns in older adults with ADHD is limited. Further research is needed to fully understand the patterns of health seeking behaviour among adult females and males as they age with a diagnosis of ADHD.

Our study findings have important policy implications. The finding that females with ADHD tended to use more healthcare services in the primary care setting, for mental health and in emergency departments offers the opportunity to target specific interventions to support and tailor their healthcare needs. Initiatives to raise awareness of the detrimental effects of delayed and/or unrecognized ADHD diagnoses in both sexes with an emphasis on the underutilization of healthcare services by males with ADHD in outpatient settings will help to improve access and potentially detect diagnoses earlier. There needs to be sex-specific considerations in ADHD policy development and implementation starting with a re-evaluation of the diagnostic assessment of ADHD symptoms in both females and males across the lifespan as the diagnosis of ADHD is not sex-equity based [47,64–67]. More research is needed to better understand the reasons behind these disparities in healthcare use among females and males with ADHD and the factors that contribute to these sex differences in order to prevent poor health outcomes in the ADHD population.

This study has strengths. We used a longitudinal retrospective cohort study (2014–2023) of linked administrative healthcare databases over a long observation period of 9 years, covering several years of the COVID-19 pandemic. Healthcare administrative databases reflect real-world clinical practices within the general population of Ontario, Canada, and provide readily available data in an electronic format which was used to reliably study trends in healthcare visits over time [68]. This is also the first study to use a validated health administrative data algorithm to identify the ADHD cohort [2]. We identified all the eligible ADHD patients and controls from the province of Ontario thereby minimizing the risk of selection bias [68]. This study involved just under one million ADHD cases and matched controls, representing a large population of Ontario residents. Matching allowed us to improve study efficiency, minimize measurable confounders (e.g., age, sex, residence, socioeconomic status), and, importantly, enabled a clinician-friendly presentation of crude visit rates between female and male ADHD cases and their respective controls during the study period, which offers greater clarity to clinicians than alternative statistical methods [69]. Also, the longitudinal nature of this study is well suited for examining annual healthcare visits, and age group is a time-varying covariate, where ADHD cases and controls are allowed to exit at any time point due to loss of follow up, death or fiscal year end.

This study also has limitations. We presented descriptive data on population trends for females and males with and without ADHD where we matched ADHD cases to controls on some key characteristics. However, healthcare administrative databases are limited in their ability to provide individual-level social and clinical characteristics such as ethnicity, gender, severity of illness, and other determinants of health [68]. Additionally, we did not assess COVID-19 diagnoses as a covariate and/or possible confounder because we did not have access to SARS-CoV-2 polymerase chain laboratory test results via the Ontario Laboratories Information System database, recognizing that the validity of diagnostic codes in outpatient claims is poor [70]. Although we measured indicators of comorbidity burden such as aggregated diagnosis groups and resource utilization band, they were only used to describe the study cohorts. We acknowledge that there may be residual confounding due to unmeasured and/or unobservable confounders that were not accounted for in this study [68]. We did not explore the reasons underlying the healthcare visit for each outcome as such information is not readily available in health administrative databases. These data may not be generalizable to different health systems and where pandemic-related restrictions may have differed. We captured insured physician outpatient visits, and it is possible that those with ADHD sought uninsured/private health care services and/or services from non-physicians.

## Conclusions

Individuals with ADHD had higher healthcare utilizations, particularly in mental health support which far exceed those of the general population. Specifically, there are substantial differences in outpatient healthcare visits among females and males with ADHD which have changed over time. These patterns can guide policy and care provision for the sex-specific needs of the ADHD population. Acknowledging and understanding these sex-specific differences is crucial for informing policymakers, healthcare providers and decision-makers to develop targeted interventions that provide more tailored and effective care. Future research needs to focus on reasons for attendance in different outpatient settings among females and males with ADHD to better understand these disparities.

## Supporting information

S1 Appendix List of OHIP outpatient service codes for family physician visits. (DOCX)

S2 Appendix List of diagnostic codes for outpatient mental health visits. (DOCX)

S1 Tables Rate differences in healthcare visits between cases and controls: A. Family physician visits, B. Mental health visits, C. Emergency department visits. The fiscal year for the Ontario Health Insurance Plan starts on April 1^st^ and ends on March 31^st^ of the following year.
(DOCX)

S1 Fig Visit rate differences among females and males with ADHD by other age groups. Data for S1 Fig are found in the Supporting information file, see S2 Tables.
(DOCX)

S2 Tables Rate differences in healthcare visits between cases and controls: A. Family physician visits, B. Mental health visits, C. Emergency department visits. The fiscal year for the Ontario Health Insurance Plan starts on April 1^st^ and ends on March 31^st^ of the following year.
(DOCX)
