# Supplementary material for: Healthcare use in individuals with and without attention-deficit/hyperactivity disorder: A population-based longitudinal matched cohort study
Source: PLOS Ment Health. 2025 Jul 28;2(7):e0000342. doi: 10.1371/journal.pmen.0000342 (PMC12798465; doi:10.1371/journal.pmen.0000342)
Supplement: S1 Appendix — (DOCX) [file pmen.0000342.s001.docx]

**S1 Appendix. List of OHIP outpatient service codes for family physician visits**

| **Service Codes** |
| --- |
| A001, A002, A003, A004, A005, A007, A008, A101, A102, A071, A131, A134, A624, A680, A888, A901, A903, A911, A912, A920, K002, K003, K004, K005, K007, K008, K013, K014, K015, K017, K022, K028, K030, K032, K033, K037, K039, K130, K131, K132, K133, K623, K680, P003, P004, P005, P008, K080, K081, K082, K087, K088, K089 |
