# Supplementary material for: Healthcare use in individuals with and without attention-deficit/hyperactivity disorder: A population-based longitudinal matched cohort study
Source: PLOS Ment Health. 2025 Jul 28;2(7):e0000342. doi: 10.1371/journal.pmen.0000342 (PMC12798465; doi:10.1371/journal.pmen.0000342)
Supplement: S2 Appendix — (DOCX) [file pmen.0000342.s002.docx]

**S2 Appendix. List of diagnostic codes for outpatient mental health visits**

| **Code** | **Diagnosis** |
| --- | --- |
| 291 | Alcoholic psychosis, delirium tremens, Korsakov's psychosis |
| 292 | Drug psychosis |
| 295 | Schizophrenia |
| 296 | Manic-depressive psychoses, involutional melancholia |
| 297 | Other paranoid states |
| 298 | Other psychoses |
| 299 | Child Psychoses (e.g. Autism) |
| 300 | Anxiety neurosis, hysteria, neurasthenia, obsessive-compulsive neurosis, reactive depression |
| 301 | Personality disorders |
| 302 | Sexual deviations |
| 303 | Alcoholism |
| 304 | Drug dependence |
| 305 | Tobacco abuse |
| 306 | Psychosomatic illness |
| 307 | Habit spasms, tics, stuttering, tension headaches, anorexia nervosa, sleep disorders, enuresis |
| 309 | Adjustment reaction |
| 311 | Depressive disorder |
| 313 | Behaviour disorders of childhood and adolescence |
| 314 | Hyperkinetic syndrome of childhood |
| 315 | Specified delays in development (eg dyslexia) |
| 897 | Economic problems |
| 898 | Marital difficulties |
| 899 | Parent-child problems |
| 900 | Problems with aged parents or in-laws |
| 901 | Family disruption/divorce |
| 902 | Education problems |
| 904 | Social maladjustment |
| 905 | Occupational problems |
| 906 | Legal problems |
| 909 | Other problems of social adjustment |
