# Supplementary material for: Healthcare use in individuals with and without attention-deficit/hyperactivity disorder: A population-based longitudinal matched cohort study
Source: PLOS Ment Health. 2025 Jul 28;2(7):e0000342. doi: 10.1371/journal.pmen.0000342 (PMC12798465; doi:10.1371/journal.pmen.0000342)
Supplement: S1 Tables — The fiscal year for the Ontario Health Insurance Plan starts on April 1st and ends on March 31st of the following year. (DOCX) [file pmen.0000342.s003.docx]

**S1 Tables. Rate differences in healthcare visits between cases and controls**

**A. Family physician visits**

| **Fiscal year** | **Age group (years)** | **Mean visits in cases per person**  **(95% CI)** | | **Mean visits in controls per person**  **(95% CI)** | | **Case-control**  **rate difference per person**  **(95% CI)** | |
| --- | --- | --- | --- | --- | --- | --- | --- |
|  |  | **Females** | **Males** | **Females** | **Males** | **Females** | **Males** |
| <2020 | 1-17 | 1.70 (1.69, 1.71) | 1.30 (1.29, 1.31) | 0.98 (0.97, 0.99) | 0.80 (0.80, 0.81) | 0.72 (0.70, 0.73) | 0.50 (0.49, 0.51) |
| <2020 | 18 and older | 7.30 (7.28, 7.32) | 6.14 (6.12, 6.15) | 4.15 (4.14, 4.16) | 2.98 (2.97, 2.99) | 3.15 (3.13, 3.17) | 3.16 (3.14, 3.18) |
| 2020 | 1-17 | 1.66 (1.65, 1.67) | 1.06 (1.05, 1.06) | 0.66 (0.65, 0.67) | 0.45 (0.45, 0.46) | 1.01 (0.99, 1.02) | 0.60 (0.60, 0.61) |
| 2020 | 18 and older | 8.24 (8.22, 8.26) | 6.34 (6.33, 6.36) | 4.12 (4.10, 4.13) | 2.76 (2.75, 2.77) | 4.12 (4.10, 4.15) | 3.59 (3.57, 3.60) |
| 2021 | 1-17 | 1.72 (1.71, 1.73) | 1.13 (1.13, 1.14) | 0.76 (0.75, 0.76) | 0.52 (0.52, 0.52) | 0.96 (0.95, 0.97) | 0.61 (0.61, 0.62) |
| 2021 | 18 and older | 8.25 (8.23, 8.26) | 6.47 (6.46, 6.48) | 4.25 (4.24, 4.26) | 2.95 (2.94, 2.96) | 4.00 (3.98, 4.02) | 3.52 (3.50, 3.53) |
| 2022 | 1-17 | 1.73 (1.73, 1.74) | 1.20 (1.19, 1.21) | 0.82 (0.81, 0.83) | 0.64 (0.64, 0.64) | 0.91 (0.90, 0.92) | 0.56 (0.55, 0.57) |
| 2022 | 18 and older | 7.33 (7.32, 7.35) | 6.14 (6.13, 6.16) | 4.03 (4.02, 4.04) | 2.91 (2.90, 2.91) | 3.31 (3.29, 3.32) | 3.24 (3.22, 3.25) |

The fiscal year for the Ontario Health Insurance Plan starts on April 1st and ends on March 31st of the following year.

**B. Mental health visits**

| **Fiscal year** | **Age group (years)** | **Mean visits in cases per person**  **(95% CI)** | | **Mean visits in controls per person**  **(95% CI)** | | **Case-control**  **rate difference per person**  **(95% CI)** | |
| --- | --- | --- | --- | --- | --- | --- | --- |
|  |  | **Females** | **Males** | **Females** | **Males** | **Females** | **Males** |
| <2020 | 1-17 | 2.30 (2.29, 2.31) | 2.09 (2.09, 2.10) | 0.22 (0.22, 0.23) | 0.13 (0.13, 0.13) | 2.08 (2.07, 2.09) | 1.97 (1.96, 1.97) |
| <2020 | 18 and older | 5.16 (5.15, 5.18) | 4.94 (4.92, 4.95) | 1.02 (1.02, 1.03) | 0.94 (0.94, 0.95) | 4.14 (4.13, 4.16) | 4.00 (3.98, 4.01) |
| 2020 | 1-17 | 2.90 (2.89, 2.91) | 2.13 (2.13, 2.14) | 0.23 (0.23, 0.24) | 0.12 (0.12, 0.12) | 2.66 (2.65, 2.68) | 2.02 (2.01, 2.02) |
| 2020 | 18 and older | 6.26 (6.25, 6.28) | 5.40 (5.39, 5.42) | 1.17 (1.17, 1.18) | 0.99 (0.98, 0.99) | 5.09 (5.07, 5.11) | 4.41 (4.40, 4.43) |
| 2021 | 1-17 | 2.69 (2.68, 2.71) | 2.03 (2.02, 2.03) | 0.27 (0.27, 0.28) | 0.12 (0.12, 0.12) | 2.42 (2.41, 2.43) | 1.90 (1.90, 1.91) |
| 2021 | 18 and older | 6.06 (6.05, 6.08) | 5.24 (5.22, 5.25) | 1.13 (1.13, 1.14) | 0.99 (0.98, 0.99) | 4.93 (4.92, 4.94) | 4.25 (4.24, 4.26) |
| 2022 | 1-17 | 2.48 (2.47, 2.49) | 1.88 (1.87, 1.89) | 0.25 (0.25, 0.26) | 0.11 (0.11, 0.11) | 2.23 (2.22, 2.24) | 1.77 (1.76, 1.78) |
| 2022 | 18 and older | 5.06 (5.05, 5.08) | 4.74 (4.73, 4.76) | 0.99 (0.99, 1.00) | 0.91 (0.91, 0.92) | 4.07 (4.06, 4.09) | 3.83 (3.82, 3.85) |

The fiscal year for the Ontario Health Insurance Plan starts on April 1st and ends on March 31st of the following year.

**C. Emergency department visits**

| **Fiscal year** | **Age group (years)** | **Mean visits in cases per person**  **(95% CI)** | | **Mean visits in controls per person**  **(95% CI)** | | **Case-control**  **rate difference per person**  **(95% CI)** | |
| --- | --- | --- | --- | --- | --- | --- | --- |
|  |  | **Females** | **Males** | **Females** | **Males** | **Females** | **Males** |
| <2020 | 1-17 | 0.84 (0.83, 0.85) | 0.49 (0.49, 0.50) | 0.42 (0.42, 0.43) | 0.32 (0.32, 0.33) | 0.42 (0.41, 0.43) | 0.17 (0.17, 0.18) |
| <2020 | 18 and older | 0.79 (0.78, 0.79) | 0.70 (0.69, 0.70) | 0.45 (0.45, 0.45) | 0.37 (0.37, 0.38) | 0.34 (0.33, 0.35) | 0.32 (0.32, 0.33) |
| 2020 | 1-17 | 0.65 (0.64, 0.65) | 0.36 (0.36, 0.36) | 0.29 (0.29, 0.30) | 0.21 (0.21, 0.22) | 0.35 (0.35, 0.36) | 0.14 (0.14, 0.15) |
| 2020 | 18 and older | 0.65 (0.64, 0.65) | 0.61 (0.60, 0.61) | 0.36 (0.36, 0.36) | 0.31 (0.31, 0.32) | 0.29 (0.28, 0.29) | 0.29 (0.29, 0.30) |
| 2021 | 1-17 | 0.75 (0.74, 0.75) | 0.43 (0.42, 0.43) | 0.37 (0.36, 0.37) | 0.27 (0.27, 0.28) | 0.38 (0.37, 0.39) | 0.15 (0.15, 0.16) |
| 2021 | 18 and older | 0.69 (0.68, 0.69) | 0.62 (0.62, 0.63) | 0.40 (0.40, 0.41) | 0.35 (0.35, 0.36) | 0.28 (0.28, 0.29) | 0.27 (0.26, 0.27) |
| 2022 | 1-17 | 0.75 (0.74, 0.75) | 0.46 (0.46, 0.46) | 0.39 (0.39, 0.40) | 0.31 (0.31, 0.31) | 0.35 (0.35, 0.36) | 0.15 (0.15, 0.16) |
| 2022 | 18 and older | 0.64 (0.64, 0.64) | 0.59 (0.59, 0.60) | 0.41 (0.41, 0.41) | 0.35 (0.35, 0.35) | 0.23 (0.22, 0.23) | 0.24 (0.24, 0.25) |

The fiscal year for the Ontario Health Insurance Plan starts on April 1st and ends on March 31st of the following year.
