# Supplementary material for: Healthcare use in individuals with and without attention-deficit/hyperactivity disorder: A population-based longitudinal matched cohort study
Source: PLOS Ment Health. 2025 Jul 28;2(7):e0000342. doi: 10.1371/journal.pmen.0000342 (PMC12798465; doi:10.1371/journal.pmen.0000342)
Supplement: S1 Fig — Data for S1 Fig are found in the Supporting information file, see S2 Tables. (DOCX) [file pmen.0000342.s004.docx]

**S1 Fig. Visit rate differences among females and males with ADHD by other age groups**
