# Supplementary material for: Healthcare use in individuals with and without attention-deficit/hyperactivity disorder: A population-based longitudinal matched cohort study
Source: PLOS Ment Health. 2025 Jul 28;2(7):e0000342. doi: 10.1371/journal.pmen.0000342 (PMC12798465; doi:10.1371/journal.pmen.0000342)
Supplement: S2 Tables — The fiscal year for the Ontario Health Insurance Plan starts on April 1st and ends on March 31st of the following year. (DOCX) [file pmen.0000342.s005.docx]

**S2 Tables. Rate differences in healthcare visits between cases and controls for other age groups**

**A. Family physician visits**

| **Fiscal year** | **Age group**  **(years)** | **Mean visits in cases per person**  **(95% CI)** | | **Mean visits in controls per person**  **(95% CI)** | | **Case-control**  **rate difference per person**  **(95% CI)** | |
| --- | --- | --- | --- | --- | --- | --- | --- |
|  |  | **Females** | **Males** | **Females** | **Males** | **Females** | **Males** |
| **<2020** | 1-9 | 0.76 (0.75, 0.77) | 0.74 (0.74, 0.75) | 0.38 (0.37, 0.38) | 0.38 (0.38, 0.39) | 0.38 (0.37, 0.40) | 0.36 (0.35, 0.37) |
|  | 10-17 | 2.35 (2.33, 2.37) | 1.86 (1.85, 1.87) | 1.40 (1.39, 1.41) | 1.22 (1.22, 1.23) | 0.95 (0.93, 0.97) | 0.64 (0.62, 0.65) |
|  | 18-29 | 6.53 (6.51, 6.56) | 4.97 (4.95, 4.99) | 3.70 (3.68, 3.72) | 2.38 (2.36, 2.39) | 2.83 (2.80, 2.86) | 2.60 (2.57, 2.62) |
|  | 30-49 | 7.26 (7.24, 7.29) | 6.80 (6.77, 6.82) | 3.81 (3.79, 3.83) | 2.92 (2.91, 2.94) | 3.45 (3.42, 3.48) | 3.88 (3.85, 3.91) |
|  | 50 and older | 9.04 (9.00, 9.09) | 8.24 (8.20, 8.28) | 5.82 (5.79, 5.86) | 5.04 (5.01, 5.08) | 3.22 (3.17, 3.28) | 3.19 (3.14, 3.25) |
| **2020** | 1-9 | 0.82 (0.81, 0.83) | 0.64 (0.64, 0.65) | 0.18 (0.17, 0.18) | 0.18 (0.18, 0.18) | 0.64 (0.63, 0.65) | 0.46 (0.46, 0.47) |
|  | 10-17 | 2.24 (2.23, 2.26) | 1.48 (1.47, 1.49) | 0.99 (0.98, 1.00) | 0.73 (0.73, 0.74) | 1.26 (1.24, 1.28) | 0.75 (0.74, 0.76) |
|  | 18-29 | 7.41 (7.38, 7.43) | 4.94 (4.93, 4.96) | 3.56 (3.54, 3.58) | 2.03 (2.02, 2.04) | 3.85 (3.82, 3.88) | 2.92 (2.90, 2.94) |
|  | 30-49 | 8.16 (8.13, 8.19) | 7.10 (7.08, 7.13) | 3.82 (3.80, 3.84) | 2.78 (2.77, 2.80) | 4.34 (4.31, 4.37) | 4.32 (4.29, 4.35) |
|  | 50 and older | 10.43 (10.39, 10.48) | 9.00 (8.96, 9.05) | 6.11 (6.07, 6.14) | 5.11 (5.08, 5.14) | 4.33 (4.27, 4.38) | 3.89 (3.84, 3.95) |
| **2021** | 1-9 | 0.69 (0.68, 0.70) | 0.62 (0.61, 0.62) | 0.20 (0.19, 0.20) | 0.17 (0.17, 0.17) | 0.50 (0.49, 0.51) | 0.45 (0.44, 0.45) |
|  | 10-17 | 2.39 (2.38, 2.41) | 1.67 (1.66, 1.68) | 1.12 (1.11, 1.13) | 0.89 (0.88, 0.90) | 1.27 (1.25, 1.29) | 0.78 (0.77, 0.80) |
|  | 18-29 | 7.32 (7.30, 7.34) | 5.07 (5.06, 5.09) | 3.63 (3.62, 3.65) | 2.26 (2.25, 2.27) | 3.69 (3.66, 3.71) | 2.81 (2.79, 2.83) |
|  | 30-49 | 8.39 (8.36, 8.41) | 7.20 (7.17, 7.22) | 4.05 (4.04, 4.07) | 2.93 (2.91, 2.94) | 4.33 (4.31, 4.36) | 4.27 (4.24, 4.29) |
|  | 50 and older | 10.50 (10.45, 10.54) | 9.28 (9.24, 9.33) | 6.45 (6.42, 6.49) | 5.41 (5.37, 5.44) | 4.04 (3.99, 4.10) | 3.88 (3.82, 3.93) |
| **2022** | 1-9 | 0.74 (0.73, 0.75) | 0.68 (0.68, 0.69) | 0.28 (0.27, 0.29) | 0.25 (0.25, 0.26) | 0.46 (0.45, 0.47) | 0.43 (0.42, 0.44) |
|  | 10-17 | 2.36 (2.35, 2.37) | 1.75 (1.74, 1.76) | 1.16 (1.15, 1.17) | 1.05 (1.04, 1.06) | 1.20 (1.18, 1.22) | 0.70 (0.69, 0.71) |
|  | 18-29 | 6.07 (6.05, 6.09) | 4.65 (4.64, 4.67) | 3.33 (3.32, 3.34) | 2.18 (2.17, 2.19) | 2.74 (2.72, 2.76) | 2.47 (2.45, 2.49) |
|  | 30-49 | 7.66 (7.64, 7.68) | 6.88 (6.86, 6.90) | 3.93 (3.92, 3.95) | 2.90 (2.89, 2.91) | 3.72 (3.70, 3.75) | 3.98 (3.95, 4.00) |
|  | 50 and older | 10.33 (10.29, 10.37) | 9.25 (9.21, 9.30) | 6.43 (6.40, 6.46) | 5.47 (5.44, 5.51) | 3.90 (3.85, 3.95) | 3.78 (3.73, 3.83) |

The fiscal year for the Ontario Health Insurance Plan starts on April 1st and ends on March 31st of the following year.

**B. Mental health visits**

| **Fiscal year** | **Age group**  **(years)** | **Mean visits in cases per person**  **(95% CI)** | | **Mean visits in controls per person**  **(95% CI)** | | **Case-control**  **rate difference per person**  **(95% CI)** | |
| --- | --- | --- | --- | --- | --- | --- | --- |
|  |  | **Females** | **Males** | **Females** | **Males** | **Females** | **Males** |
| **<2020** | 1-9 | 1.56 (1.54, 1.57) | 1.68 (1.67, 1.69) | 0.04 (0.03, 0.04) | 0.05 (0.05, 0.05) | 1.52 (1.51, 1.54) | 1.63 (1.62, 1.64) |
|  | 10-17 | 2.82 (2.80, 2.84) | 2.51 (2.50, 2.52) | 0.35 (0.35, 0.36) | 0.21 (0.20, 0.21) | 2.47 (2.45, 2.49) | 2.31 (2.29, 2.32) |
|  | 18-29 | 4.34 (4.32, 4.36) | 3.84 (3.82, 3.86) | 0.87 (0.86, 0.88) | 0.71 (0.70, 0.71) | 3.48 (3.45, 3.50) | 3.13 (3.11, 3.15) |
|  | 30-49 | 5.66 (5.63, 5.68) | 6.06 (6.04, 6.09) | 1.07 (1.06, 1.08) | 1.18 (1.17, 1.20) | 4.58 (4.56, 4.61) | 4.88 (4.85, 4.91) |
|  | 50 and older | 5.91 (5.87, 5.94) | 5.68 (5.64, 5.72) | 1.25 (1.24, 1.27) | 1.09 (1.08, 1.11) | 4.66 (4.62, 4.69) | 4.58 (4.55, 4.62) |
| **2020** | 1-9 | 1.96 (1.94, 1.98) | 1.71 (1.71, 1.72) | 0.03 (0.03, 0.04) | 0.04 (0.04, 0.05) | 1.93 (1.91, 1.94) | 1.67 (1.66, 1.68) |
|  | 10-17 | 3.55 (3.53, 3.57) | 2.57 (2.55, 2.58) | 0.37 (0.37, 0.38) | 0.19 (0.19, 0.20) | 3.17 (3.15, 3.19) | 2.37 (2.36, 2.39) |
|  | 18-29 | 5.62 (5.60, 5.64) | 4.23 (4.22, 4.25) | 0.99 (0.98, 1.00) | 0.71 (0.70, 0.72) | 4.63 (4.60, 4.65) | 3.52 (3.50, 3.54) |
|  | 30-49 | 6.59 (6.57, 6.61) | 6.55 (6.53, 6.58) | 1.23 (1.22, 1.24) | 1.25 (1.24, 1.26) | 5.36 (5.34, 5.39) | 5.31 (5.28, 5.33) |
|  | 50 and older | 7.11 (7.07, 7.15) | 6.30 (6.27, 6.34) | 1.51 (1.49, 1.53) | 1.24 (1.23, 1.26) | 5.60 (5.56, 5.64) | 5.06 (5.02, 5.10) |
| **2021** | 1-9 | 1.52 (1.50, 1.53) | 1.53 (1.52, 1.54) | 0.03 (0.03, 0.03) | 0.04 (0.04, 0.05) | 1.49 (1.47, 1.50) | 1.49 (1.48, 1.50) |
|  | 10-17 | 3.47 (3.45, 3.49) | 2.55 (2.53, 2.56) | 0.43 (0.42, 0.44) | 0.20 (0.20, 0.21) | 3.04 (3.02, 3.06) | 2.34 (2.33, 2.35) |
|  | 18-29 | 5.42 (5.40, 5.43) | 4.06 (4.05, 4.08) | 0.97 (0.96, 0.97) | 0.70 (0.69, 0.71) | 4.45 (4.43, 4.47) | 3.36 (3.35, 3.38) |
|  | 30-49 | 6.57 (6.55, 6.59) | 6.36 (6.34, 6.38) | 1.19 (1.19, 1.20) | 1.24 (1.23, 1.25) | 5.37 (5.35, 5.40) | 5.12 (5.09, 5.14) |
|  | 50 and older | 6.64 (6.60, 6.67) | 6.19 (6.15, 6.22) | 1.45 (1.44, 1.47) | 1.27 (1.25, 1.28) | 5.19 (5.15, 5.23) | 4.92 (4.88, 4.96) |
| **2022** | 1-9 | 1.39 (1.38, 1.40) | 1.41 (1.40, 1.41) | 0.03 (0.03, 0.04) | 0.04 (0.04, 0.04) | 1.35 (1.34, 1.37) | 1.36 (1.36, 1.37) |
|  | 10-17 | 3.17 (3.15, 3.18) | 2.39 (2.38, 2.40) | 0.39 (0.38, 0.40) | 0.18 (0.18, 0.19) | 2.78 (2.76, 2.80) | 2.20 (2.19, 2.21) |
|  | 18-29 | 4.20 (4.18, 4.21) | 3.58 (3.56, 3.59) | 0.82 (0.81, 0.82) | 0.63 (0.62, 0.63) | 3.38 (3.37, 3.40) | 2.95 (2.94, 2.97) |
|  | 30-49 | 5.63 (5.62, 5.65) | 5.77 (5.76, 5.79) | 1.07 (1.07, 1.08) | 1.14 (1.14, 1.15) | 4.56 (4.54, 4.58) | 4.63 (4.61, 4.65) |
|  | 50 and older | 6.16 (6.13, 6.19) | 5.83 (5.80, 5.86) | 1.30 (1.29, 1.32) | 1.23 (1.22, 1.25) | 4.86 (4.83, 4.89) | 4.60 (4.57, 4.64) |

The fiscal year for the Ontario Health Insurance Plan starts on April 1st and ends on March 31st of the following year.

**C. Emergency department visits**

| **Fiscal year** | **Age group**  **(years)** | **Mean visits in cases per person**  **(95% CI)** | | **Mean visits in controls per person**  **(95% CI)** | | **Case-control**  **rate difference per person**  **(95% CI)** | |
| --- | --- | --- | --- | --- | --- | --- | --- |
|  |  | **Females** | **Males** | **Females** | **Males** | **Females** | **Males** |
| **<2020** | 1-9 | 0.59 (0.58, 0.60) | 0.44 (0.43, 0.44) | 0.33 (0.32, 0.34) | 0.29 (0.29, 0.30) | 0.26 (0.25, 0.27) | 0.15 (0.14, 0.15) |
|  | 10-17 | 1.10 (1.09, 1.11) | 0.61 (0.60, 0.61) | 0.49 (0.48, 0.50) | 0.35 (0.35, 0.36) | 0.61 (0.59, 0.62) | 0.25 (0.25, 0.26) |
|  | 18-29 | 0.89 (0.88, 0.90) | 0.68 (0.67, 0.69) | 0.47 (0.46, 0.48) | 0.35 (0.34, 0.35) | 0.42 (0.41, 0.43) | 0.33 (0.33, 0.34) |
|  | 30-49 | 0.79 (0.78, 0.79) | 0.77 (0.76, 0.78) | 0.44 (0.44, 0.45) | 0.40 (0.39, 0.40) | 0.34 (0.33, 0.35) | 0.38 (0.37, 0.39) |
|  | 50 and older | 0.70 (0.69, 0.71) | 0.71 (0.70, 0.73) | 0.42 (0.41, 0.43) | 0.41 (0.40, 0.42) | 0.28 (0.27, 0.30) | 0.31 (0.29, 0.32) |
| **2020** | 1-9 | 0.45 (0.44, 0.46) | 0.29 (0.28, 0.29) | 0.21 (0.21, 0.22) | 0.17 (0.17, 0.18) | 0.24 (0.23, 0.25) | 0.11 (0.11, 0.12) |
|  | 10-17 | 0.85 (0.84, 0.86) | 0.48 (0.47, 0.49) | 0.35 (0.34, 0.35) | 0.26 (0.25, 0.26) | 0.50 (0.49, 0.51) | 0.22 (0.22, 0.23) |
|  | 18-29 | 0.69 (0.69, 0.70) | 0.59 (0.58, 0.59) | 0.36 (0.36, 0.37) | 0.28 (0.28, 0.29) | 0.33 (0.32, 0.34) | 0.30 (0.30, 0.31) |
|  | 30-49 | 0.67 (0.67, 0.68) | 0.67 (0.67, 0.68) | 0.36 (0.35, 0.36) | 0.33 (0.33, 0.34) | 0.32 (0.31, 0.33) | 0.34 (0.33, 0.35) |
|  | 50 and older | 0.57 (0.56, 0.58) | 0.62 (0.61, 0.63) | 0.35 (0.35, 0.36) | 0.36 (0.35, 0.37) | 0.21 (0.20, 0.23) | 0.26 (0.24, 0.27) |
| **2021** | 1-9 | 0.56 (0.55, 0.57) | 0.37 (0.36, 0.37) | 0.28 (0.28, 0.29) | 0.24 (0.23, 0.24) | 0.27 (0.26, 0.29) | 0.13 (0.12, 0.13) |
|  | 10-17 | 0.94 (0.94, 0.95) | 0.54 (0.54, 0.55) | 0.42 (0.42, 0.43) | 0.31 (0.31, 0.32) | 0.52 (0.51, 0.53) | 0.23 (0.22, 0.23) |
|  | 18-29 | 0.76 (0.75, 0.76) | 0.61 (0.61, 0.62) | 0.42 (0.41, 0.42) | 0.32 (0.32, 0.33) | 0.34 (0.33, 0.35) | 0.29 (0.28, 0.30) |
|  | 30-49 | 0.69 (0.68, 0.70) | 0.68 (0.67, 0.69) | 0.39 (0.39, 0.40) | 0.37 (0.37, 0.38) | 0.30 (0.29, 0.31) | 0.31 (0.30, 0.32) |
|  | 50 and older | 0.62 (0.61, 0.63) | 0.63 (0.62, 0.64) | 0.39 (0.39, 0.40) | 0.41 (0.40, 0.41) | 0.23 (0.22, 0.24) | 0.22 (0.21, 0.24) |
| **2022** | 1-9 | 0.63 (0.62, 0.63) | 0.44 (0.44, 0.45) | 0.34 (0.33, 0.35) | 0.29 (0.29, 0.30) | 0.28 (0.27, 0.30) | 0.15 (0.14, 0.16) |
|  | 10-17 | 0.90 (0.89, 0.91) | 0.53 (0.53, 0.54) | 0.43 (0.42, 0.43) | 0.32 (0.32, 0.33) | 0.47 (0.46, 0.48) | 0.21 (0.20, 0.21) |
|  | 18-29 | 0.69 (0.69, 0.70) | 0.58 (0.58, 0.59) | 0.43 (0.42, 0.43) | 0.33 (0.33, 0.34) | 0.26 (0.26, 0.27) | 0.25 (0.24, 0.26) |
|  | 30-49 | 0.64 (0.64, 0.65) | 0.64 (0.64, 0.65) | 0.40 (0.39, 0.40) | 0.36 (0.35, 0.36) | 0.25 (0.24, 0.26) | 0.28 (0.28, 0.29) |
|  | 50 and older | 0.60 (0.59, 0.61) | 0.62 (0.61, 0.63) | 0.40 (0.39, 0.41) | 0.40 (0.40, 0.41) | 0.20 (0.18, 0.21) | 0.22 (0.20, 0.23) |

The fiscal year for the Ontario Health Insurance Plan starts on April 1st and ends on March 31st of the following year.
